# Effect of Extraction Method and Ethanol Concentration on the Antioxidant and α-Glucosidase Inhibitory Activities of *Hornstedtia scyphifera* var. *fusiformis* Shoots:LC-HRMS/MS Profiling of the Antioxidant-Optimized Extract

**DOI:** 10.3390/molecules31213941

**Published:** 2026-11-01

**Authors:** Silvia Indri Yati, Rika Hartati, Irda Fidrianny

**Affiliations:** Department of Pharmaceutical Biology, School of Pharmacy, Institut Teknologi Bandung, Bandung 40132, Indonesia; 30726011@mahasiswa.itb.ac.id or indriyatisilvia@gmail.com (S.I.Y.); rikahar@itb.ac.id (R.H.)

**Keywords:** *Hornstedtia scyphifera*, ethanol extraction, antioxidant activity, α-glucosidase inhibition, LC-HRMS/MS

## Abstract

Despite its traditional use in Sumatra, *Hornstedtia scyphifera* var. *fusiformis* (Zingiberaceae) has not been evaluated for antioxidant or α-glucosidase inhibitory activity in its shoots. Vegetative shoot extracts were prepared by maceration and reflux using 70% and 96% ethanol and assessed for total phenolic content (TPC), total flavonoid content (TFC), antioxidant activity (DPPH, CUPRAC, FRAP, ABTS), and α-glucosidase inhibitory activity. 96% ethanol extracts showed higher TPC, TFC, and antioxidant activity than 70% ethanol; maceration in 96% ethanol (M96) gave the highest TPC (138.87 ± 9.29 mg GAE/g) and CUPRAC activity (106.62 ± 1.59 mg AEAC/g). Reflux with 70% ethanol (R70), however, showed markedly stronger α-glucosidase inhibition (6811.94 ± 528.27 mg AEAGIC/g) than the other extracts despite low phenolic and flavonoid content. α-Glucosidase inhibition did not follow phenolic, flavonoid, or antioxidant parameters. In chemometric analysis (PCA, HCA) the extracts were separated mainly according to the ethanol concentration rather than extraction technique. LC-HRMS/MS profiling of the antioxidant-optimized M96 extract putatively annotated 20 secondary metabolites, led by the flavonol ermanin and the sesquiterpenoid nootkatone. Because only M96 was profiled, the compounds underlying R70’s α-glucosidase inhibition remain unidentified; extraction conditions thus affect the two activities differently, but the chemical basis of R70’s activity requires further investigation.

## 1. Introduction

Indonesia is one of the world’s major centers of Zingiberaceae diversity. Yet phytochemical and pharmacological work on *Hornstedtia* has lagged far behind better-studied genera such as *Zingiber*, *Curcuma*, and *Alpinia*.

*Hornstedtia scyphifera* (J.Koenig) Steud. var. *fusiformis* Holttum, known locally in West Sumatra as “Sijangkang,” is reported as endemic to Sumatra [1,2]. What is known of its chemistry so far comes almost entirely from its suspended roots and rhizome: these organs contain phenolics, flavonoids, saponins, triterpenoids, steroids, and alkaloids, and their extracts show antioxidant activity (DPPH IC_50_ = 51.89 mg/L for the methanol root extract), antibacterial action against *Staphylococcus aureus* and *Escherichia coli*, and anti-inflammatory effects in a rat model [1,2,3]. None of this prior work looked beyond a single antioxidant assay (DPPH), examined the vegetative shoots, or considered antidiabetic activity. A separate line of work using UHPLC-HRMS on *H. scyphifera* leaves (variety unspecified) found terpenoids, flavonoids, and phenolics as the dominant compound classes, including two previously undescribed sesquiterpenoids [4]. Taken together, these studies leave the vegetative shoots of var. *fusiformis*, along with its antidiabetic potential and the effect of extraction conditions on either activity, essentially untouched.

Other *Hornstedtia* species provide some evidence of antidiabetic potential in the genus. Leaf extracts of *Hornstedtia conoidea* reached total phenolic and flavonoid contents of 314.69 mg GAE/g and 43.62 mg QE/g with a DPPH IC_50_ of 14.21 µg/mL [5], and methylated kaempferol derivatives isolated from several Malaysian *Hornstedtia* species inhibited both DPPH radicals and α-glucosidase: 3-methylkaempferol alone gave IC_50_ values of 78.6 µM and 21.0 µM, respectively [6]. These findings suggest that flavonol-type compounds may contribute to both activities in the genus, although this has been shown for isolated compounds rather than for crude extracts and remains to be tested in other species.

Solvent polarity and extraction technique are well known to shape how much phenolic and flavonoid material is recovered from a plant, and with it, the antioxidant and antidiabetic activity of the resulting extract. Maceration and reflux with ethanol at different concentrations remain popular choices for this reason (they are simple to run and cover a useful range of polarities), but no one has yet asked how these parameters affect the bioactivity of *H. scyphifera*. This study addresses that question directly: we evaluated how extraction method (maceration vs. reflux) and ethanol concentration (70% vs. 96%) affect the total phenolic content, total flavonoid content, antioxidant activity (DPPH, CUPRAC, FRAP, ABTS), and α-glucosidase inhibitory activity of *H. scyphifera* var. *fusiformis* vegetative shoots, and used exploratory correlation analysis and chemometrics, together with LC-HRMS/MS profiling of the antioxidant-optimized extract, to characterize the extracts.

## 2. Results

### 2.1. Extraction Yield

Yields for the four extraction conditions are given in Table 1. Reflux with 70% ethanol (R70) gave the highest yield at 10.72%, followed by maceration with 70% ethanol (M70, 7.50%), reflux with 96% ethanol (R96, 5.59%), and maceration with 96% ethanol (M96, 4.74%). The pattern held regardless of extraction technique: 70% ethanol extracted more total mass than 96% ethanol in both cases.

### 2.2. Total Phenolic and Total Flavonoid Content

TPC and TFC values for the four extracts are shown in Table 2. TPC ranged from 28.31 ± 0.79 mg GAE/g in R70 to 138.87 ± 9.29 mg GAE/g in M96, and TFC ranged from 3.13 ± 0.29 to 12.15 ± 0.52 mg QE/g across the same two extremes. One-way ANOVA indicated significant differences among the four extracts for both parameters (*p* < 0.05; Section 4.9). M96 stood out with the highest TPC and TFC, followed in order by R96, M70, and R70 in both TPC and TFC.

### 2.3. Antioxidant Activity

Antioxidant capacity was evaluated by two complementary mechanisms: radical scavenging (DPPH and ABTS) and metal ion reducing power (FRAP, Fe^3+^ to Fe^2+^; CUPRAC, Cu^2+^ to Cu^+^). Antioxidant activity across the four assays is summarized in Table 2. DPPH activity was highest in R96 (137.89 ± 2.04 mg AEAC/g), well above M96 (98.23 ± 5.64 mg AEAC/g); M70 and R70 trailed both and did not differ from each other (88.03 ± 6.09 and 86.31 ± 1.58 mg AEAC/g, respectively). CUPRAC followed a related but not identical order: M96 came out on top (106.62 ± 1.59 mg AEAC/g), ahead of R96, M70, and R70, and here all four extracts differed significantly from one another (*p* < 0.05).

FRAP activity was again highest in R96 (76.88 ± 5.43 mg AEAC/g), with M70 and R70 statistically indistinguishable at the bottom (45.21 ± 1.68 and 47.32 ± 0.77 mg AEAC/g). For ABTS, M96 and R96 were comparable to each other (60.10 ± 4.32 and 56.18 ± 5.29 mg AEAC/g) and both clearly ahead of M70 and R70, which again did not differ from one another (40.11 ± 0.79 and 37.52 ± 1.10 mg AEAC/g).

### 2.4. Alpha-Glucosidase Inhibitory Activity

Alpha-glucosidase inhibitory activity, expressed as acarbose equivalents, is also reported in Table 2, and here the pattern breaks sharply from the other assays. R70 reached 6811.94 ± 528.27 mg AEAGIC/g, five to seven times higher than the other three extracts, which clustered between 916.32 ± 88.68 and 1320.09 ± 436.70 mg AEAGIC/g and were not significantly different from each other. Tukey’s test indicated that R70 differed from all three extracts (*p* < 0.05). AEAGIC values above 1000 mg/g indicate stronger inhibition than an equal mass of acarbose. This finding should be interpreted in the context of the enzyme source, as acarbose is a potent inhibitor of mammalian intestinal α-glucosidases but only a weak inhibitor of yeast (*S. cerevisiae*) α-glucosidase [7]. AEAGIC values are therefore assay-specific: they allow comparison among extracts tested under identical conditions but do not indicate that R70 is more potent than acarbose against mammalian α-glucosidases or in vivo.

### 2.5. Correlation Analysis

Because the extracts separated mainly according to ethanol concentration in the chemometric analysis (Section 2.6), Pearson correlations among TPC, TFC, and the five activity parameters were examined separately within the 70% and 96% ethanol extracts, rather than pooling all four conditions together (Figure 1). Because each group consisted of technical replicates of only two extracts, the coefficients mainly reflect the differences between these extracts; the correlations are therefore treated as exploratory and were not tested for statistical significance. In the resulting heatmaps, correlation coefficients range from −1 (blue) to +1 (red), with redder hues denoting stronger positive relationships and bluer tones denoting negative ones. Correlations spanning moderate to very strong emerge among several parameters, but the overall pattern differs sharply between the two ethanol concentrations, and for a handful of parameter pairs the relationship even changes direction. Within the 70% ethanol extracts (Figure 1A), TPC, CUPRAC, and ABTS form a tightly inter-correlated cluster, in line with earlier reports that these electron-transfer-based assays tend to track the phenolic content of a plant extract. Alpha-glucosidase inhibitory activity sits apart from this cluster and moves in the opposite direction, most notably against TPC, suggesting that within this concentration group the extracts richest in phenolics are not necessarily the ones best able to inhibit the enzyme. That picture flips within the 96% ethanol extracts (Figure 1B): α-glucosidase inhibitory activity now aligns positively with TPC, TFC, and CUPRAC, while DPPH and FRAP break away to form a separate cluster running in the opposite direction. Whether these differences reflect a shift in the extracted constituents cannot be determined from the present data (Section 3).

### 2.6. Chemometric Analysis

Principal Component Analysis (PCA) and Hierarchical Cluster Analysis (HCA) were used to look for multivariate structure in the TPC, TFC, and activity data. The PCA score plot separated the four extracts, with PC1 and PC2 accounting for 71.2% and 16.8% of total variance (Figure 2A). PC1 split the 70% ethanol extracts (M70, R70), which sat on the negative side, from the 96% ethanol extracts (M96, R96) on the positive side, reflecting a smaller difference associated with the extraction method. Notably, M70 and R70 clustered closely despite their marked difference in α-glucosidase inhibition. Since TPC, TFC, and the four antioxidant parameters differed mainly with ethanol concentration, whereas α-glucosidase inhibition followed a different pattern (Table 2), the separation in the score plot appears to be driven mainly by the antioxidant-related parameters; the PCA therefore does not capture the variance underlying the activity of R70. Replicates for each extract clustered tightly together, reflecting analytical repeatability rather than reproducibility of extraction.

The HCA dendrogram told the same story: two main clusters corresponding to 70% and 96% ethanol, each of which split further into maceration and reflux sub-branches (Figure 2B). As in the PCA, replicates within each extract grouped closely.

### 2.7. LC-HRMS/MS Compound Identification

M96, which obtained the highest composite ranking score (Section 4.5) and represents the antioxidant-optimized extract, was selected for LC-HRMS/MS profiling; R70, which showed the strongest α-glucosidase inhibition, was not profiled. This profiling turned up a broad range of secondary metabolites; identification was restricted to features with a mzCloud similarity score above 90%, a threshold met by 52 features, of which 20 were classified as secondary metabolites after the exclusion of compounds unlikely to be of plant origin. All annotations are tentative (MSI level 2), as they were not confirmed with authentic reference standards. Table 3 lists the top 10 by peak area, spanning several metabolite classes: flavonoids (ermanin, catechin, isokaempferide, quercitrin), sesquiterpenoids (nootkatone, caryophyllene oxide), monoterpenoids (carvone, α-pinene-2-oxide), an alkaloid (trigonelline), and a phenolic acid (4-coumaric acid). Ermanin showed the largest peak area, followed by nootkatone and caryophyllene oxide; together these three compounds clearly dominated the identified secondary metabolite pool, while the remaining 7 compounds contributed comparatively minor peak areas. A broad range of secondary metabolites was tentatively annotated in *H. scyphifera* through LC-HRMS/MS profiling (Figure 3). The prominent peak at 14.17 min eluted at the end of the high-organic wash and the onset of column re-equilibration (Section 4.5) and was not included in Table 3 because no compound feature was detected at this retention time in the processed data set (the latest annotated feature eluted at 11.89 min); its origin, possibly related to the system or the gradient change, could not be determined. The intense peaks between approximately 9.4 and 10.2 min corresponded mainly to lipid-related features (e.g., N-acylethanolamines, monoacylglycerols, and ceramides), which are not listed in Table 3 because the table is restricted to secondary metabolites.

## 3. Discussion

The higher extraction yield obtained with 70% ethanol (M70, R70: 7.50–10.72%) compared to 96% ethanol (M96, R96: 4.74–5.59%) is consistent with the general principle that more aqueous solvents co-extract a broader range of polar constituents, including sugars and other hydrophilic material, alongside the target compounds, thereby increasing total extract mass [5]. A comparable yield range has been reported for *Hornstedtia havilandii* rhizome, leaf, and fruit macerated in water (7.98–10.87%) [8]. Similar solvent-dependent yield trends have also been documented outside the genus: aqueous and ethanolic maceration of *Zingiber officinale* rhizome gave differing yields depending on solvent polarity [9], red ginger (*Z. officinale* var. *rubrum*) macerated in 96% ethanol showed a comparable order of magnitude [10], and a recent multi-solvent screen of *Etlingera dostseiana* (Zingiberaceae) similarly linked higher yields to more aqueous ethanol [11]. Together, these reports suggest that yields in the 5–11% range are typical for ethanol-based maceration and reflux of Zingiberaceae tissue, and that the lower yields obtained with 96% ethanol in the present study likely reflect exclusion of the hydrophilic fraction, concentrating the resulting extract toward medium-polarity constituents such as flavonoid aglycones and terpenoids. The additional mass in the 70% ethanol extracts, which was not accompanied by higher phenolic and flavonoid contents, likely consisted largely of polar non-phenolic material such as free sugars, oligosaccharides, and partially soluble polysaccharides, together with proteins and peptides, organic acids, and inorganic salts, which are abundant in young vegetative tissues and dilute the phenolic and flavonoid contents expressed per gram of extract.

TPC and TFC were consistently highest in the 96% ethanol extracts, particularly M96 (138.87 mg GAE/g; 12.15 mg QE/g), consistent with reports that ethanol-water systems with lower water content more efficiently solubilize medium-polarity phenolic acids and flavonoid aglycones [5]. This value falls below the TPC reported for *H. conoidea* methanolic leaf extract (314.69 mg GAE/g) [5] and for *Hedychium* species leaves from the Philippines, which reached comparably high TPC and TFC in ethanolic extraction [12], but well above a separate ethanolic *H. conoidea* leaf extract (13.29 mg GAE/g) [13] and above the phenolic content reported for *Etlingera dostseiana* leaves and rhizomes [11]. A similarly wide range has been reported outside Zingiberaceae in a related family-level comparison, where ethanolic leaf extracts of purple-orange sweet potato reached TPC values an order of magnitude higher than their tuber counterparts depending on solvent and plant part [14]. A similar solvent- and region-dependent spread of TPC/TFC values has also been reported within a single Zingiberaceae species: red ginger rhizomes collected from seven locations in Java varied several-fold in TPC and TFC depending on growing region [15], and methanolic fractionation of red ginger likewise gave TPC values that shifted markedly with solvent polarity [10]. This range of reported values, spanning roughly one to two orders of magnitude even within the same genus, underscores how strongly TPC and TFC depend on solvent, plant part, and possibly geographic origin. Because the correlation structure itself differed between ethanol concentrations (Section 2.5), the relationships discussed below are considered separately for the 70% and 96% ethanol extracts rather than pooled together. TFC and CUPRAC correlated positively within both concentration groups (r = 0.53 in the 70% ethanol extracts, r = 0.61 in the 96% ethanol extracts), consistent with the widely reported role of flavonoids as effective donors in both single-electron-transfer (CUPRAC) and mixed hydrogen-atom-transfer/electron-transfer (ABTS) antioxidant mechanisms [5,12,13,16]. TPC’s relationship with DPPH, however, differed sharply between the two groups: weakly positive in the 70% ethanol extracts (r = 0.38) but strongly negative in the 96% ethanol extracts (r = −0.95). These exploratory correlations may reflect differences in the constituents accompanying the phenolics at each ethanol concentration; however, without LC-HRMS/MS data for the 70% ethanol extracts, this explanation remains speculative. The differing rank order between DPPH (highest in R96) and CUPRAC (highest in M96) is also consistent with the distinct redox mechanisms these assays probe: DPPH and ABTS are generally classified as mixed hydrogen-atom-transfer/electron-transfer assays, whereas CUPRAC and FRAP operate as single-electron-transfer assays and are more responsive to compounds with lower standard reduction potentials [16]. This is consistent with a recent statistical evaluation of DPPH, ABTS, FRAP, and Folin–Ciocalteu assays across plant matrices, which likewise found that these assays do not necessarily rank samples identically, precisely because they probe different antioxidant mechanisms [17]. Extracts with comparable phenolic content can therefore rank differently across assays depending on the redox properties of their dominant constituents, a pattern also noted in a recent critical review of spectrophotometric antioxidant methods [18].

The most notable finding of this study is the sharply elevated α-glucosidase inhibitory activity of R70 (6811.94 mg AEAGIC/g), five- to seven-fold higher than the other three extracts despite its comparatively low TPC and TFC. This pattern runs counter to that reported for methylated kaempferol derivatives isolated from other Malaysian *Hornstedtia* species, where the most potent antioxidant compound (3-methylkaempferol) was also the most potent α-glucosidase inhibitor (IC_50_ = 78.6 µM and 21.0 µM, respectively) [6], and to a report of an α-glucosidase-inhibitory essential oil from *H. scyphifera* flowers (IC_50_ = 21.44 µg/mL) [19]. A broader survey of Vietnamese Zingiberaceae species similarly found that most rhizome extracts with strong α-glucosidase inhibition were rich in flavonoids, including isokaempferide isolated from *Zingiber zerumbet* [20] (the same flavonol identified in the present LC-MS/MS profile), while a phenylbutenoid-guided study of cassumunar ginger rhizome reported an even more potent α-glucosidase IC_50_ of 6.3 µg/mL [21], and a leaf polyphenol extract of *Zingiber roseum* gave a comparatively modest IC_50_ of 570 µg/mL despite containing 16 LC-MS/MS-identified phenolics [22] (a species for which other constituents such as β-sitosterol have separately been linked to antidiabetic activity via PPARγ modulation [23], consistent with a broader review noting that *Z. roseum* produces a chemically diverse set of antidiabetic-relevant metabolites beyond its phenolic fraction [24]), while an aqueous rhizome extract of *Z. officinale* gave an IC_50_ of 581.4 µg/mL alongside moderate DPPH and FRAP activity [25]. Collated across these five comparisons, the genus- and family-level pattern is that flavonol-rich Zingiberaceae extracts generally combine antioxidant and antidiabetic activity, which does not predict the divergence between the two activities observed for R70 in the present study. This divergence suggests that the activity of R70 is largely attributable to non-flavonoid constituents. The aluminum chloride assay mainly quantifies flavones and flavonols, so other inhibitor classes, such as terpenoids, polar non-phenolic metabolites, or compounds formed or released during heating under reflux, would not be reflected in TFC values; moreover, activities reported for isolated compounds cannot be directly extrapolated to crude extracts, in which synergistic or antagonistic interactions may occur. A straightforward chemical explanation is difficult to establish from the present data, however, because LC-MS/MS profiling was performed only on M96, not on R70 itself. M96 was selected for this analysis on the basis of a composite ranking score weighted toward antioxidant capacity (Section 4.5); consequently, the present data do not allow the activity of R70 to be linked to specific chemical constituents. M96’s chemistry was led by the flavonol ermanin, followed by the sesquiterpenoids nootkatone and caryophyllene oxide, yet M96’s own α-glucosidase inhibition (1320.09 ± 436.70 mg AEAGIC/g) was statistically indistinguishable from M70 and R96 and only about one-fifth that of R70 (6811.94 ± 528.27 mg AEAGIC/g). The one extract confirmed to contain nootkatone and caryophyllene oxide therefore does not display an activity level approaching R70’s, despite separate reports that sesquiterpene-derived scaffolds, including synthetic nootkatone-thiazole hybrids [26] and β-caryophyllene itself [27,28], can inhibit α-glucosidase or improve glucose metabolism through non-phenolic mechanisms. If anything, this pattern argues against rather than for a simple terpenoid-based explanation, and points instead to either an unmeasured difference in R70’s own chemical makeup or the involvement of compounds not captured by profiling M96 alone. The concentration-specific correlation analysis (Section 2.5) adds a further layer to this picture: within the 70% ethanol extracts, α-glucosidase inhibition correlated strongly and negatively with TPC and CUPRAC, a pattern driven largely by R70’s combination of low phenolic content and outlying enzyme-inhibitory activity relative to M70. Within the 96% ethanol extracts, by contrast, α-glucosidase inhibition correlated positively with TPC, TFC, and CUPRAC. Because each of these correlations rests on technical replicates of only two extracts, they should be read as exploratory, but the reversal itself suggests that the relationship between phenolic/flavonoid content and α-glucosidase inhibition is not fixed across the two ethanol concentrations, and that R70’s extreme value has an outsized influence on the correlation pattern specific to the 70% ethanol extracts. Identifying the constituents responsible for the activity of R70 will require LC-HRMS/MS profiling and bioassay-guided fractionation of this extract.

The dominance of PC1 (71.2% of variance) in separating extracts by ethanol concentration rather than extraction method parallels a near-identical finding in a chemometric study of elephant ginger (*Zingiber officinale* var. *officinale*) extracts, where PC1 accounted for 93.3% of variance and similarly separated samples primarily by ethanol concentration [29], a pattern the same research group linked to solvent polarity when optimizing extraction conditions for this species [30]. PCA/HCA-based separation of extracts primarily by solvent or origin rather than processing method has also been reported for *Graptophyllum pictum* (Acanthaceae), where hierarchical clustering of phenolic and antioxidant data formed distinct solvent-based clusters [31], and for Anatolian propolis, where PCA and HCA jointly classified samples by geographic origin using combined phenolic and antioxidant datasets [32]. These reports suggest that solvent polarity may influence extract composition more strongly than extraction technique; in the present study, however, each condition was represented by a single extract analyzed in technical replicates, so this observation should be confirmed with independent extraction replicates.

Considered individually against the wider literature, most of the ten tentatively identified compounds carry some precedent for antioxidant or antidiabetic relevance, although the strength of that precedent varies considerably from one compound to the next. Because none of these assignments were confirmed here through isolation, in silico modelling, or HPLC co-elution with reference standards, they are discussed below as tentative candidates on the basis of activities reported for the pure compounds and cannot be assumed to account for the α-glucosidase inhibition of R70.

Ermanin, the compound with the largest peak area, is a methylated flavonol structurally close to the kaempferol derivatives already linked to antioxidant and α-glucosidase activity in other *Hornstedtia* species [6]; direct pharmacological data on ermanin itself remain scarce, and this comparison should therefore be read as a structural analogy rather than direct evidence. Nootkatone, the second-largest peak, is better supported: it activates AMPK and suppresses ERK signalling to improve glucose metabolism in diabetic mice [33], and separately reduces oxidative stress markers in a doxorubicin-induced cardiac injury model [34], although its contribution to the present extracts was not assessed. Caryophyllene oxide, if its annotation is confirmed, would also be of interest: it occurs as a major essential-oil constituent alongside strong antioxidant activity in *Stevia rebaudiana* (Asteraceae) [35], and structurally related caryophyllene-derived meroterpenoids act as potent, non-competitive α-glucosidase inhibitors [36], consistent with the antidiabetic activity already reported for its parent compound β-caryophyllene [27].

Catechin needs little introduction as an antioxidant, but it is also a demonstrated α-glucosidase inhibitor that enhances glucose uptake in adipocyte models [37], although its contribution to the present extracts was not assessed. Isokaempferide offers the most direct precedent of any compound on this list: it is chemically identical to kaempferol-3-O-methyl ether, which was the single most potent α-glucosidase inhibitor among the sesquiterpenes and flavonoids isolated from *Zingiber zerumbet* (IC_50_ = 7.88 µM) [38], and has itself been isolated from the same species [20]. Carvone is one of the few compounds here evaluated as a pure substance rather than inferred from extract-level data: a recent study measured its antioxidant capacity across six complementary assays and confirmed α-amylase and α-glucosidase inhibitory activity directly [39]. Trigonelline, familiar as a coffee alkaloid, has arguably the deepest literature base of any compound identified here: a dedicated mechanistic review describes how it lowers blood glucose by enhancing insulin secretion, reducing oxidative stress, and improving glucose tolerance and insulin resistance [40].

Alpha-pinene-2-oxide is structurally related to α-pinene, which shows hypoglycaemic and hypolipidaemic activity in alloxan-induced diabetic rats [41] and contributes, together with limonene, to the α-glucosidase-inhibitory activity of *Pistacia lentiscus* essential oil [42]. Quercitrin (quercetin-3-O-rhamnoside), a widely distributed flavonol glycoside, has been reported to possess both antioxidant and α-glucosidase inhibitory activities [43,44]. 4-Coumaric acid closes the list: in streptozotocin-induced diabetic rats it lowers blood glucose and gluconeogenic enzyme activity while upregulating pancreatic GLUT2 expression [45], and structural differences among cinnamic-acid-backbone phenolic acids have been shown to alter carbohydrate-digesting enzyme inhibition [46]; as a minor M96 constituent, however, it is unlikely to explain the activity of R70.

While the integrated in vitro and LC-HRMS/MS approaches used in this study provided valuable insights, several limitations remain, including the use of a single extract per condition analyzed in technical replicates (without independent extraction replicates), the restriction of LC-HRMS/MS profiling to the antioxidant-optimized M96 extract, the lack of in vivo validation, the putative (rather than confirmed) identification of bioactive metabolites, the limited sampling scope (single plant part and location), and the absence of mechanism of action and toxicity assessments. Future studies should therefore focus on profiling and bioassay-guided fractionation of the R70 extract using independent extraction replicates, isolating and structurally confirming the key active compounds, evaluating their efficacy and safety through in vivo and clinical models, and elucidating their molecular mechanisms of action to fully establish the therapeutic potential of *H. scyphifera* as an antidiabetic and antioxidant agent.

## 4. Materials and Methods

### 4.1. Chemicals and Reagents

All chemicals and reagents used in this study were of analytical or HPLC grade and were obtained from Merck (Darmstadt, Germany) or Sigma-Aldrich (St. Louis, MO, USA). Ethanol, HPLC-grade methanol, and 0.1% trifluoroacetic acid (TFA) were used for extraction and chromatographic analysis. Folin–Ciocalteu reagent, aluminium chloride, sodium acetate, acetic anhydride, gallic acid, and quercetin were used for total phenolic and flavonoid content determination. 2,2-Diphenyl-1-picrylhydrazyl (DPPH), 2,2′-azino-bis(3-ethylbenzothiazoline-6-sulfonic acid) (ABTS), potassium persulfate, iron (III) chloride, 2,4,6-tripyridyl-s-triazine (TPTZ), copper (II) chloride, neocuproine, ammonium acetate, and ascorbic acid were used for the antioxidant activity assays (DPPH, ABTS, FRAP, and CUPRAC). Alpha-glucosidase from *Saccharomyces cerevisiae*, p-nitrophenyl-α-D-glucopyranoside (pNPG), 0.1 M phosphate buffer (pH 6.8), sodium carbonate (Na_2_CO_3_), and acarbose (positive control) were used for the α-glucosidase inhibitory activity assay. Hydrochloric acid and sodium phosphate served as supporting reagents throughout the analytical procedures.

### 4.2. Instrumentation

Sample preparation and extraction used an analytical balance (Mettler Toledo^®^, Greifensee, Switzerland), micropipettes (Eppendorf^®^, Hamburg, Germany), and a rotary evaporator (Büchi Rotavapor^®^ R-100, Büchi Labortechnik, Flawil, Switzerland). High-performance liquid chromatography (HPLC) was carried out on a Shimadzu HPLC system (Shimadzu Corporation, Kyoto, Japan), and liquid chromatography–tandem mass spectrometry (LC–MS/MS) on a Thermo Scientific™ Vanquish™ UHPLC system coupled to an Orbitrap™ Exploris 240 high-resolution mass spectrometer (Thermo Fisher Scientific, Waltham, MA, USA). The DPPH, ABTS, FRAP, and CUPRAC assays were read on a UV-Visible spectrophotometer (Thermo Fisher Scientific, Waltham, MA, USA), while the α-glucosidase inhibitory assay was read on a Multiskan SkyHigh microplate spectrophotometer (Thermo Scientific^®^, Waltham, MA, USA) using 96-well microplates (Corning^®^, Corning, NY, USA).

### 4.3. Plant Material and Sample Preparation

Vegetative shoots of *Hornstedtia scyphifera* var. *fusiformis* were collected from their natural habitat in Simpang Sugiran Village, Guguak District, Lima Puluh Kota Regency, West Sumatra, Indonesia, in April 2026. The plant material was identified and authenticated, and a voucher specimen (No. 2397/IT1.C11.2/TA.00/2026) was deposited at the Herbarium Bandungense, School of Life Sciences and Technology (SITH), Institut Teknologi Bandung, Indonesia. Samples were oven-dried and ground into powder prior to extraction.

### 4.4. Extraction Procedure

Dried powder (300 g) was extracted by maceration and reflux, each carried out in three extraction cycles (successive extractions of the same plant material, with the solvent replaced by fresh solvent in each cycle). Maceration ran for 24 h per cycle at room temperature; reflux ran for 2 h per cycle. Both methods used ethanol at 70% and 96%, with a sample-to-solvent ratio of approximately 1:3 (*w*/*v*) per cycle (cumulative ratio 1:9); solvent renewal was applied to maintain the concentration gradient and minimize solvent saturation, and the same ratio was used for all conditions to allow direct comparison. The filtrates of the three cycles were pooled into a single extract per condition, concentrated under reduced pressure at 40–50 °C, dried further at 40 °C, and stored in sealed amber containers. Yield was calculated as the mass of dried extract relative to the initial dry powder weight.

### 4.5. LC-HRMS/MS Profiling and Compound Identification

The extract subjected to LC-HRMS/MS profiling was selected using a simple additive ranking method: each of the four extracts was ranked from 1 (lowest) to 4 (highest) on every measured parameter (extraction yield, TPC, TFC, the four antioxidant assays, and α-glucosidase inhibitory activity), and the ranks were summed into a composite score. M96 obtained the highest composite score (26 out of a possible 32) and was therefore selected for LC-HRMS/MS analysis. Because seven of the eight parameters (yield, TPC, TFC, and the four antioxidant assays) are unrelated to α-glucosidase inhibition and each parameter received equal weight, this score was inherently weighted toward antioxidant capacity; M96 therefore represents the antioxidant-optimized extract rather than the extract with the strongest α-glucosidase inhibitory activity.

The M96 extract was analyzed on a Thermo Scientific™ Vanquish™ Horizon UHPLC coupled to an Orbitrap™ Exploris 240 high-resolution mass spectrometer (Thermo Fisher Scientific, Bremen, Germany). Separation used a Thermo Scientific™ Accucore™ Phenyl Hexyl column (100 × 2.1 mm, 2.6 µm) at 40 °C, with water (A) and acetonitrile (B), both containing 0.1% formic acid, under a gradient of 5–50% B (0–8 min), 50–98% B (8–9 min), held at 98% B (9–12 min), then returned to 5% B (12–15 min), at 0.3 mL/min. Prior to analysis, the extract (1 mg) was dissolved in 1 mL of 80% MS-grade methanol, vortexed at 3000 rpm for 2 min, and filtered through a 0.20 µm nylon filter into an HPLC vial. Injections of 5 µL were run in positive ESI mode via an OptaMax™ NG H-ESI source (Thermo Fisher Scientific, Bremen, Germany), with a full-MS scan range of *m*/*z* 67–1000 at 60,000 FWHM resolution, followed by data-dependent MS2 (dd-MS2) at 15,000 FWHM with normalized collision energies of 30, 50, and 150. Compounds were identified by accurate mass, and MS/MS fragmentation was matched against the mzCloud, mzVault, NIST HRMS, and ChemSpider databases. Compounds were tentatively annotated (MSI level 2) when the mzCloud similarity score exceeded 90% and the mass error was within 5 ppm. The LC-HRMS/MS analysis was performed by PT EBM Saintifik dan Teknologi (MarkHerb), Bandung, Indonesia.

### 4.6. Determination of Total Phenolic and Total Flavonoid Content

Total phenolic content (TPC) was determined by the Folin–Ciocalteu method [47]. A 50 µL aliquot of extract (10,000 µg/mL) was combined with 500 µL of 10% Folin–Ciocalteu reagent and 400 µL of 1 M Na_2_CO_3_ and left to react for 30 min; absorbance was read at 765 nm in six replicates. Gallic acid (60–130 µg/mL) served as the standard, and TPC was expressed as mg gallic acid equivalent per gram of extract (mg GAE/g).

Total flavonoid content (TFC) was determined by the aluminium chloride method [48]. A 100 µL aliquot of extract was combined with 300 µL methanol, 20 µL of 10% AlCl_3_, 20 µL of 1 M sodium acetate, and 560 µL distilled water, then incubated for 30 min; absorbance was read at 415 nm in six replicates. Quercetin (40–110 µg/mL) served as the standard, and TFC was expressed as mg quercetin equivalent per gram of extract (mg QE/g).

### 4.7. Antioxidant Activity Assays

#### 4.7.1. DPPH Radical Scavenging Assay

DPPH radical scavenging activity was measured using a modified method [49]. A 12.5 µL aliquot of extract (from a 10,000 µg/mL stock) was diluted with methanol to 125 µL, mixed with 750 µL of 50 µg/mL DPPH solution, and left in the dark for 30 min; absorbance was read at 517 nm in six replicates. Ascorbic acid (10–40 µL from a 200 µg/mL stock, six concentrations in triplicate) provided the calibration curve (R^2^ ≥ 0.99), and activity was expressed as mg ascorbic acid equivalent antioxidant capacity per gram of extract (mg AEAC/g).

#### 4.7.2. ABTS Radical Cation Decolorization Assay

The ABTS•^+^ radical cation was generated by combining 4000 µg/mL ABTS diammonium salt with 700 µg/mL potassium persulfate [50,51], incubating for 30 min at room temperature, then storing at 4 °C for 24 h in the dark. The stock was diluted with ethanol to an absorbance of 0.71 ± 0.2 at 734 nm. Extract (12.5 µL from a 30,000 µg/mL stock) was diluted with ethanol to 500 µL, mixed with 500 µL of the ABTS working solution, and kept in the dark with no further incubation; absorbance was read at 734 nm in six replicates, with ascorbic acid as the standard.

#### 4.7.3. FRAP Assay

The FRAP reagent was prepared from 5380 ppm FeCl_3_·6H_2_O, 3120 ppm TPTZ (in 0.04 M HCl), and 0.3 M acetate buffer (pH 3.6) at a 1:1:10 ratio [52]. Extract (12.5 µL from a 10,000 µg/mL stock) was diluted with distilled water to 500 µL, mixed with 500 µL FRAP reagent, and incubated in the dark for 30 min; absorbance was read at 595 nm in six replicates, with ascorbic acid as the standard.

#### 4.7.4. CUPRAC Assay

The CUPRAC reagent was prepared from CuCl_2_·2H_2_O (1705 µg/mL), neocuproine (1562 µg/mL), and ammonium acetate buffer (pH 7.0) to a 100 µg/mL working solution [52]. Extract (12.5 µL from a 10,000 µg/mL stock) was diluted with buffer to 250 µL, then mixed with 750 µL CUPRAC reagent; absorbance was read at 450 nm in six replicates, with ascorbic acid as the standard.

Ascorbic acid calibration curves for all four assays were built from at least six concentrations in triplicate, and only curves with R^2^ ≥ 0.99 were used for quantification.

### 4.8. Alpha-Glucosidase Inhibitory Activity Assay

Alpha-glucosidase inhibitory activity was assessed by a modified spectrophotometric method [53]. A 10 µL aliquot of the sample or acarbose solution (both dissolved in DMSO) was combined with 40 µL of 0.1 M phosphate buffer (pH 6.8) and 25 µL of 5 mM pNPG, and the mixture was pre-incubated for 5 min at 37 °C. The reaction was initiated by adding 25 µL of *S. cerevisiae* α-glucosidase (0.015 U/mL), giving a final reaction volume of 100 µL, and incubated for a further 15 min at 37 °C. The reaction was stopped with 100 µL of 0.2 M Na_2_CO_3_, and absorbance was read at 400 nm using a microplate reader, with acarbose as the positive control. To correct for the intrinsic absorbance of the extracts, a sample blank, in which buffer replaced the enzyme, was prepared for each sample. Percentage inhibition was calculated as:

% Inhibition = [(A_C_ − A_CB_) − (A_S_ − A_SB_)]/(A_C_ − A_CB_) × 100

where A_C_ and A_CB_ are the absorbances of the control and control blank, and A_S_ and A_SB_ are the absorbances of the sample and sample blank, respectively.

An acarbose calibration curve was constructed using acarbose solutions of 2000–6000 µg/mL; a 10 µL volume of each solution was added to the reaction, corresponding to 20–60 µg acarbose per reaction. The percentage inhibition (y, %) was plotted against the amount of acarbose per reaction (x, µg), giving y = 0.4464x + 32.43 (R^2^ = 0.9927; linear range 41.36–59.21% inhibition). The extracts were tested at 2000 µg/mL (M70 and M96), 750 µg/mL (R70), and 5000 µg/mL (R96); a 10 µL volume of each solution was added to the reaction, corresponding to 20, 20, 7.5, and 50 µg extract per reaction, respectively. These concentrations were selected so that the mean percentage inhibition of each extract fell within the linear range of the calibration curve. Because acarbose and the extracts were added at the same volume to the same final reaction volume, no additional dilution factor was applied. The percentage inhibition of each replicate was substituted as y into the regression equation to obtain the acarbose-equivalent amount (x, µg), and α-glucosidase inhibitory activity was expressed as acarbose equivalent α-glucosidase inhibitory capacity (AEAGIC, mg acarbose equivalent/g extract), calculated as AEAGIC = (x/W) × 1000, where W is the amount of extract per reaction (µg).

### 4.9. Statistical Analysis

Data were analyzed in Minitab 22. Values from six technical replicates (repeated analytical measurements of a single extract per condition) are reported as mean ± SD; normality and homogeneity of variance were checked before further testing. One-way ANOVA followed by Tukey’s post hoc test (α = 0.05) was used to compare the extracts; because the replicates were not independent extractions, the resulting differences reflect variability between extracts relative to analytical variability and do not establish the reproducibility of the extraction process. Pearson correlations among TPC, TFC, and the antioxidant/α-glucosidase activity parameters were visualized as a heatmap, calculated separately within the 70% and 96% ethanol groups (n = 12 technical measurements from two extracts per group) and interpreted as exploratory, without significance testing. MetaboAnalyst (v6.0) was used for chemometric analysis after autoscaling (mean-centering and division by the standard deviation of each variable); PCA and HCA were applied to visualize clustering patterns among *Hornstedtia scyphifera* var. *fusiformis* extracts. HCA was performed using Euclidean distance and Ward’s linkage.

## 5. Conclusions

Under the conditions tested, differences among the extracts of *H. scyphifera* var. *fusiformis* vegetative shoots were associated mainly with ethanol concentration rather than extraction technique; as each condition was represented by a single extract analyzed in technical replicates, these differences require confirmation with independent extraction replicates. The 96% ethanol extracts showed higher total phenolic and flavonoid contents and antioxidant capacity, with M96 giving the highest TPC and CUPRAC values, whereas R70 showed the strongest α-glucosidase inhibition despite its low phenolic and flavonoid contents. LC-HRMS/MS profiling of the antioxidant-optimized M96 extract putatively annotated 20 secondary metabolites, the most abundant being ermanin and nootkatone. These observations indicate that extraction conditions affected the antioxidant and α-glucosidase inhibitory activities differently and suggest that the latter may involve constituents other than phenolics and flavonoids; as R70 was not chemically profiled, these represent associations rather than established chemical causation. To the best of our knowledge, this is the first report on the phytochemical profile and the antioxidant and α-glucosidase inhibitory activities of the vegetative shoots of this plant. These findings should be regarded as preliminary in vitro results; profiling and bioassay-guided fractionation of R70, together with independent extraction replicates, are required to confirm its activity and identify the responsible constituents.

## Figures and Tables

**Figure 1:**
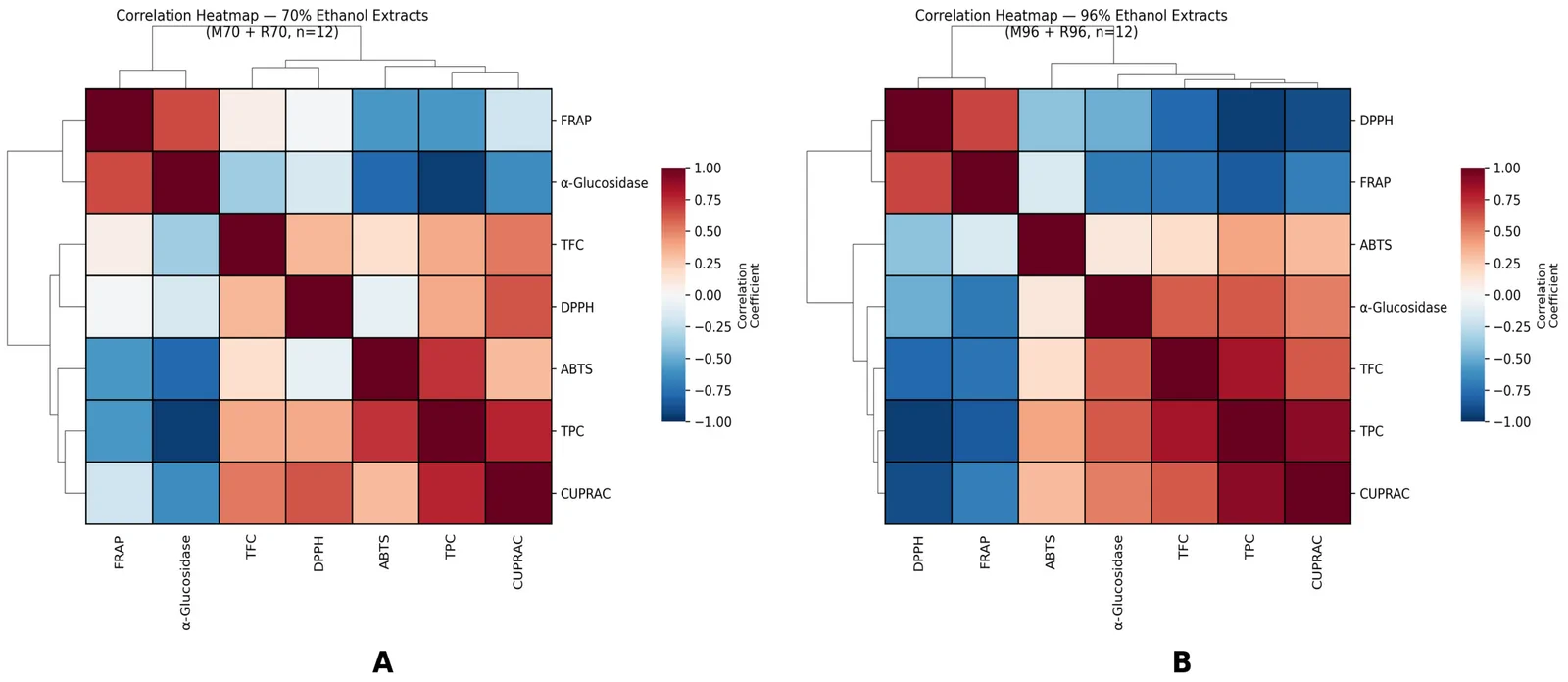
Correlation heatmaps of TPC, TFC, and antioxidant/α-glucosidase inhibitory activity assays (DPPH, CUPRAC, FRAP, ABTS, and α-glucosidase), calculated separately for (**A**) the 70% ethanol extracts (M70 and R70 pooled, n = 12) and (**B**) the 96% ethanol extracts (M96 and R96 pooled, n = 12) of *H. scyphifera*. The color scale ranges from blue (r = 1.0) to red (r = 1.0), with redder hues indicating stronger positive correlations and bluer tones indicating negative correlations. Each group comprises six technical replicates of each of the two extracts (exploratory analysis).

**Figure 2:**
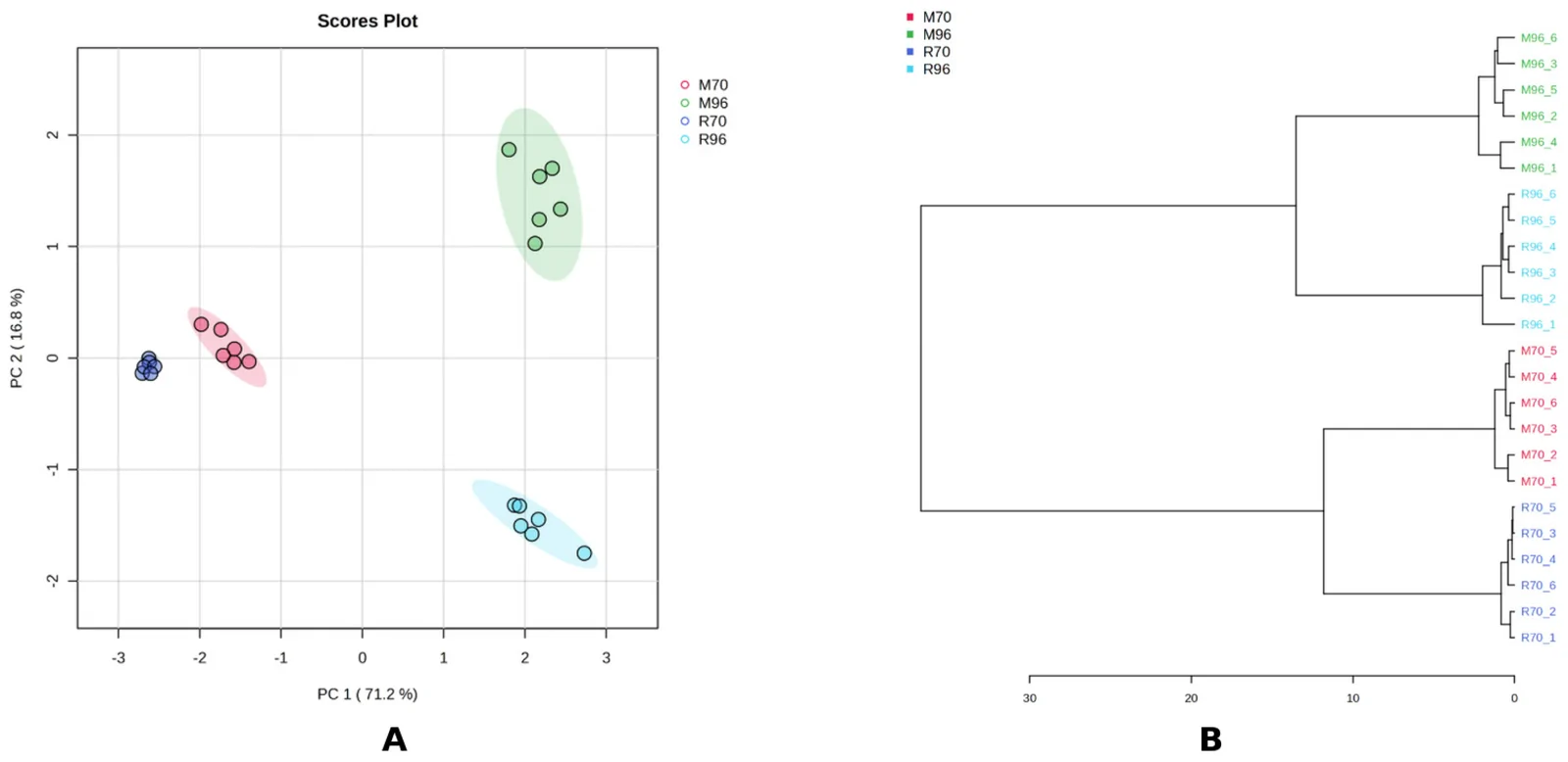
PCA score plot and HCA dendrogram of *H. scyphifera* extracts based on total phenolic content, total flavonoid content, and antioxidant/α-glucosidase inhibitory activity parameters. (**A**) PCA score plot; (**B**) HCA dendrogram. M70: 70% ethanol maceration; M96: 96% ethanol maceration; R70: 70% ethanol reflux; R96: 96% ethanol reflux. n = 6 technical replicates per extract. PCA and HCA were performed on autoscaled data; HCA used Euclidean distance and Ward’s linkage.

**Figure 3:**
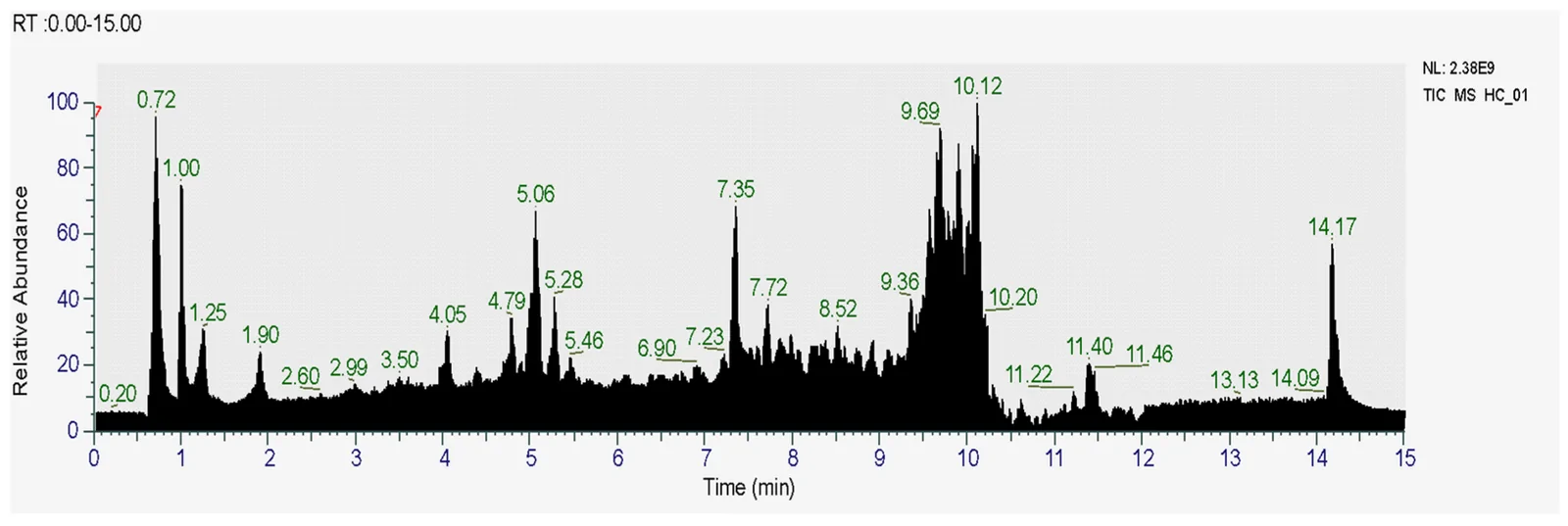
Total ion chromatogram (TIC) of the M96 extract of *H. scyphifera* var. *fusiformis* with T: 40 °C; flow rate: 0.3 mL/min; total run time: 15 min; sample: 1 mg in 1 mL (80% MeOH); injection volume: 5 µL.

**Table 1:** Extraction yield of *Hornstedtia scyphifera* var. *fusiformis* obtained by different extraction methods.

Extraction Method	Yield (%)
M70	7.50
M96	4.74
R70	10.72
R96	5.59

M70: maceration with 70% ethanol; M96: maceration with 96% ethanol; R70: reflux with 70% ethanol; R96: reflux with 96% ethanol. Extraction yield was calculated from a single combined extract obtained after three extraction cycles per condition; the three cycles were successive extractions of the same plant material with fresh solvent; no standard deviation or statistical comparison is therefore reported.

**Table 2:** Total phenolic content (TPC), total flavonoid content (TFC), antioxidant activity, and α-glucosidase inhibitory activity of *Hornstedtia scyphifera* var. *fusiformis* extracts obtained by different extraction methods.

Parameter	M70	M96	R70	R96
TPC (mg GAE/g)	36.77 ± 1.61 ^c^	138.87 ± 9.29 ^a^	28.31 ± 0.79 ^d^	57.77 ± 2.05 ^b^
TFC (mg QE/g)	3.35 ± 0.37 ^c^	12.15 ± 0.52 ^a^	3.13 ± 0.29 ^c^	10.55 ± 0.65 ^b^
DPPH (mg AEAC/g)	88.03 ± 6.09 ^c^	98.23 ± 5.64 ^b^	86.31 ± 1.58 ^c^	137.89 ± 2.04 ^a^
CUPRAC (mg AEAC/g)	52.66 ± 6.44 ^c^	106.62 ± 1.59 ^a^	45.33 ± 1.55 ^d^	93.66 ± 4.40 ^b^
FRAP (mg AEAC/g)	45.21 ± 1.68 ^c^	59.16 ± 9.67 ^b^	47.32 ± 0.77 ^c^	76.88 ± 5.43 ^a^
ABTS (mg AEAC/g)	40.11 ± 0.79 ^b^	60.10 ± 4.32 ^a^	37.52 ± 1.10 ^b^	56.18 ± 5.29 ^a^
α-Glucosidase (mg AEAGIC/g)	1020.11 ± 221.40 ^b^	1320.09 ± 436.70 ^b^	6811.94 ± 528.27 ^a^	916.32 ± 88.68 ^b^

Data are presented as mean ± SD of six technical replicates (n = 6) of a single extract per condition. ^a–d^ Different superscript letters within the same row indicate significant differences (*p* < 0.05) (one-way ANOVA, Tukey’s test). M70 = maceration with 70% ethanol; M96 = maceration with 96% ethanol; R70 = reflux with 70% ethanol; R96 = reflux with 96% ethanol. GAE = gallic acid equivalent; QE = quercetin equivalent; AEAC = ascorbic acid equivalent antioxidant capacity; AEAGIC/g = acarbose equivalent α-glucosidase inhibitory capacity (mg acarbose equivalent per g extract; values above 1000 indicate stronger inhibition than an equal mass of acarbose).

**Table 3:** LC-HRMS/MS results of the top 10 tentative secondary metabolites (by peak area) in the M96 extract of *H. scyphifera* var. *fusiformis*.

Proposed Compound	Retention Time (min)	Molecular Weight	Molecular Formula	Peak Area	Similarity (%)	Mass Error (ppm)	Metabolite Class
Ermanin	8.537	314.079	C_17_H_14_O_6_	1,296,374,473	93.2	0.93	Flavonoid (flavonol)
Nootkatone	9.581	218.167	C_15_H_22_O	853,285,246	91.5	0.65	Sesquiterpenoid
Caryophyllene oxide	7.772	220.183	C_15_H_24_O	187,822,991	92.5	0.81	Sesquiterpenoid
Catechin	3.023	290.079	C_15_H_14_O_6_	181,223,987	98.7	0.81	Flavonoid (flavan-3-ol)
Isokaempferide	6.890	300.064	C_16_H_12_O_6_	138,323,475	98.8	0.96	Flavonoid (flavonol)
Carvone	5.975	150.105	C_10_H_14_O	115,833,154	92.7	0.77	Monoterpenoid
Trigonelline	0.737	137.048	C_7_H_7_NO_2_	82,020,944	99.4	−0.39	Alkaloid
α-Pinene-2-oxide	5.241	152.120	C_10_H_16_O	54,266,051	91.5	0.50	Monoterpenoid
4-Coumaric acid	3.789	164.047	C_9_H_8_O_3_	32,717,204	99.0	0.85	Phenolic acid
Quercitrin	4.423	448.101	C_21_H_20_O_11_	25,737,604	96.6	0.50	Flavonoid (flavonol glycoside)

Compound identifications were restricted to features with a mzCloud similarity score above 90% and were assigned as Level 2 (putatively annotated compounds) according to the Metabolomics Standards Initiative (MSI) guidelines. Stereochemical descriptors are reported as assigned by the database match and could not be confirmed by mass spectrometry alone. Mass error (ppm) = (observed *m*/*z* − theoretical *m*/*z*)/theoretical *m*/*z* × 10^6^. Caryophyllene oxide and α-pinene-2-oxide were detected as [M+H−H_2_O]^+^ ions and all other compounds as [M+H]^+^ ions.

## Data Availability

Derived data supporting the findings of this study are available from the corresponding author on request.
